# NMR-based metabolomics associated with chronic kidney disease in humans and animals: a one health perspective

**DOI:** 10.1007/s11010-021-04222-1

**Published:** 2021-07-26

**Authors:** Elena Hunter, Benita Percival, Zeeshan Ahmad, Ming-Wei Chang, John A. Hunt, Séverine Tasker, Luisa De Risio, Philippe B. Wilson

**Affiliations:** 1 Nottingham Trent University, Brackenhurst Lane, Southwell, NG25 0QF UK; 2 De Montfort University, The Gateway, Leicester, LE1 9BH UK; 3 Nanotechnology and Integrated Bioengineering Centre, University of Ulster, Jordanstown Campus, Newtownabbey, Northern Ireland UK; 4 Friars Gate, Linnaeus Veterinary Limited, Solihull, B90 4BN UK

**Keywords:** Chronic kidney disease, Cat, Metabolite, Metabolomics, NMR

## Abstract

Chronic kidney disease (CKD) is a renal dysfunction that can lead to high rates of mortality and morbidity, particularly when coupled with late diagnosis. CKD has become a major health problem due to its challenging detection at early stages when clear symptoms are yet to be presented. Thus, CKD is likely to be identified when the substantive conditions of the disease are manifest. In order to address the development of the disease and provide necessary treatments at the initial stage, the investigation of new biomarkers and metabolites associated with early detection of CKD are needed. Identified metabolites could be used to confirm the presence of the disease, obtain information on its mechanism and facilitate the development of novel pharmaceutical treatments. Such metabolites may be detected from biofluids and tissues using a range of analytical techniques. There are a number of metabolites that have been identified by mass spectrometry at high sensitivities, whilst the detection of metabolites directly from biofluids using NMR could present a more rapid way to expand our understanding of this disease. This review is focused on NMR-based metabolomics associated with CKD in humans and animals.

## Introduction

Chronic kidney disease (CKD) is a progressive renal dysfunction. It raises a serious concern worldwide due to its challenging detection at early stages. CKD includes five stages calculated from either albumin or serum creatinine in urine. Stage one is characterised by estimated glomerular filtration rate (eGFR) being over 90 ml/min/1.73m^2^, whereas in stage two, eGFR is equal to 89–60 ml/min/1.73m^2^ [1, 2]. These two stages can be asymptomatic. eGFR is reduced to lower than 60 ml/min/1.73m^2^ in stage three, where electrolyte disorders and acid–base disbalance can also be observed [1]. Transplantation or dialysis are required at stage five due to eGFR being lower than 15 ml/min/1.73m^2^ [1]. This leads to an inability of the kidneys to maintain both homeostasis and healthy concentrations of metabolites in urine and blood. In order to prevent further development of CKD at the early stages of the disease, metabolites that are accumulated in blood or urine can be identified. Moreover, these metabolites could also provide insight into metabolic pathways and mechanisms that lead to the progression of the disease. This identification can be carried out by a range of analytical techniques. The most common method for the investigation of metabolites as biomarkers for CKD is mass spectrometry. Although mass spectrometry is the most common technique for such analysis of metabolites, it has several disadvantages such as the requirement of substantial sample, analytical protocol preparation and the destructive nature of the technology. Therefore, the current review is focused on metabolites associated with CKD that can be detected using NMR. In addition, this review will summarise metabolites obtained from humans and animals to provide an insight into the differences and similarities of the disease within different groups of organisms.

## Potential biomarkers for the detection of CKD

The role of metabolites in the detection of kidney failure is manifest. As healthy kidneys have an impact on circulating metabolite levels through filtration, secretion, reabsorption, and metabolism, in the case of kidney failure, metabolites that are considered to be cleared by the kidney, start to fluctuate in concentration in biofluids and tissues. Therefore, it is conceivable that the level and range of metabolites in readily accessible biofluids could help to identify CKD within its early stages.

Chen et al. analysed serum in an untargeted metabolomics workflow in 2155 participants in total including healthy controls and patients with CKD stages from one to five. As a result, five metabolites associated with CKD, 5-methoxytryptophan (5-MTP), canavanino-succinate, taurine, acetylcarnitine, and tiglylcarnitine, were identified in humans [3]. Such biomarkers could be detected at the early stage of the disease. 5-MTP was found to be strongly related to clinical markers for early stage identification of CKD. The level of 5-MTP, which is involved in endogenous tryptophan metabolism, decreases in serum but increases in urine with the progression of CKD [3]. This can be due to a reduction in reabsorption of 5-MTP and tryptophan in the kidney [3]. It was observed that the levels of canavanine and succinate were upregulated in blood samples of humans with CKD [4, 5].

Canavanine has a similar structure to arginine, but is a non-protein amino acid. If the concentration of canavanine is too high, the amino acid starts to accumulate in the blood and cells. It can affect metabolism, and inhibit the synthesis of proteins and immunoglobulins. Bedolla et al. investigated the levels of both canavanine and creatine in serum of human patients with CKD before and after dialysis [4]. It was found that the level of canavanine in the serum of patients with CKD ranged from 10 to 21 mg/dl, whilst the level of creatinine was observed to be from 3 to 14 mg/dl in the control healthy group [4]. However, dialysis decreased the levels of canavanine and creatinine to 9.5–19 and 0.9 to 7.5 mg/dl, respectively [4]. One of the criteria for analysis of renal function is the glomerular filtration rate (GFR). If GFR is decreased, albumin can reach urine that is adjusted by creatinine concentration. Therefore, a ratio of albumin-creatinine (ACR) can act as another indicator of CKD [2].

Disturbance of purine metabolism could also be linked to the progression of CKD. Such disturbances can result in the production of hypoxanthine and breakdown to xanthine and uric acid. The accumulation of uric acid in the blood could subsequently lead to hyperuricemia. According to Chuang et al., hyperuricemia occurs when the serum uric acid level is more than 7.0 mg/dl and more than 6.0 mg/dl for males and females, respectively [6]. Therefore, an increase in levels of uric acid can be an indicator of CKD in humans. Animals such as cows, several species of rats, cats, and dogs carry an enzyme called urate oxidase in their liver which is capable of converting uric acid to allantoin [7]. However, such an enzyme was not found in the liver of humans and several other primates such as the Cynomolgus monkey [7]. This means that purine catabolism ends with uric acid in humans, but, in cats and dogs, the final product is allantoin. However, excessive production of reactive oxygen species might lead to the oxidation of uric acid to allantoin in humans [8].

Allantoin is a marker of oxidative stress which was found to be associated with mortality in patients with CKD [9]. It has been reported that the concentration of allantoin in plasma in human patients with chronic renal failure was (27.1 ± 13.8) μmol/l compared to the control concentration of 4.67 ± 2.99 μmol/l [10]. Yang et al. found that the level of allantoin increases in patients with CKD from 18 ± 13.5 μg/ml (healthy patients) to 238 ± 57 μg/ml [11]. Allantoin from patients with CKD was then injected into the C57BL/6 mouse model [11] and it was observed that mice in the treatment group with allantoin had increased scratching behaviour compared to the control group at day 7 [11]. According to Yang et al. this could be because allantoin activates DRG neurons and therefore cause symptoms such as a pruritus [11]. In addition, upregulation of levels of ribothymidine and allantoin were also detected in rats with CKD [12].

Albumin and uromodulin were found to be associated with CKD [13, 14]. It has been reported that urine of healthy dogs contains low concentrations of albumin (3.1 ± 1.4 mg/dl) and a high concentration of uromodulin (11.9 ± 2.3 mg/dl), whereas the urine samples of dogs with CKD showed the opposite pattern, where the level of albumin increased to 26.6 mg/dl, but the concentration of uromodulin decreased to near 0 mg/dl [13]. Moreover, levels of uromodulin decreased in the urine of human patients with CKD from 14.17 to 1.83 mg/g but increased in serum samples of those patients [14]. Furthermore, Ferlizza et al. observed upregulation of methylguanidine, dimethylamine, and kynurenic acid metabolites in the urine of dogs with CKD using NMR [13]. Upregulation of kynurenic acid was also detected in rats with CKD [12].

Metabolic dysfunction in amino acids, fatty acids, taurine, choline was also observed elsewhere [15]. Fumarate and ribonate were upregulated in the serum of human patients with CKD [9]. Fumarate is involved in the citric acid cycle, whereas ribonate is the intermediate of the pentose phosphate pathway [9]. The levels of lactate and malate were also increased; however, urine citrate was reduced in humans with CKD [5]. Moreover, it has been reported that an induced level of adenine was found in patients with CKD [15]. A possible reason for this may be that adenine, which is metabolised to 2,8-dihydroxyadenine in vivo and insoluble in water, can affect functions of the kidney by promoting excretion of nitrogen compounds and influence the balance of electrolytes [15].

The upregulation of creatinine, fibroblast growth factor-23 (FGF-23), and SDMA were found to be associated with CKD in cats [16]. Serum creatinine increased from 1.29 mg/dl of the control to 1.99 mg/dl in cats with CKD [17]. The levels of homocysteine, which is an amino acid in S-adenosyl methionine metabolism were higher in serum of cats with CKD compared to the control healthy group. It was found that levels of homocysteine increased along with the progression of the disease. Another study has reported that levels of FGF‐23 in the plasma of cats start to increase at the early stages of CKD [18]. Moreover, uHSP72:uCr, which is urinary heat shock protein-72 could be another biomarker for the identification of CKD in cats and dogs. It was observed that the levels of uHSP72:uCr were increased in cats and dogs with CKD [16, 19].

Other metabolites were identified to be correlated with CKD. For instance, high levels of betaine, leucine, trimethylamine-N-oxide, citrate methyl histidine were increased in serum of humans compared to a control group [20]. However, the levels of glutamine, pyruvate, valine, acetate, and tyrosine were downregulated in human patients with CKD [20]. Patients with both diabetes and CKD had decreased levels of urea, acetate, arginine, pyruvate, N-acetyl-glycoprotein, creatinine, and citrate compared to patients with the single condition of CKD [20]. Conversely, increases in levels of tyrosine, valine, lactate, leucine, and choline were detected in patients with both diabetes and CKD compared to patients with CKD alone [21].

### Influence of gut microbiota on CKD

The connection between gut microbiota and CKD has been observed elsewhere [22]. It has been found that CKD can result in intestinal dysbiosis that leads to the formation of uremic toxins such as *p*-cresol sulphate and indoxyl sulphate [22]. *P*-cresol sulphate and indoxyl sulphate are produced by gut microbiota and may lead to glomerular sclerosis and tubulointerstitial fibrosis [22, 23]. These toxins could influence the progression of CKD. One study investigated a group of human patients with CKD who presented significantly higher levels of both *p*-cresol sulphate and indoxyl sulphate than a control group. Moreover, 3-Indolepropionic acid (IPA), which is a product of deamination of microbiota, was significantly lower in serum samples of humans with CKD than the control group [22]. IPA was found to be significantly related to CKD development and renal function changes [22]. Therefore, a high level of IPA leads to a lower risk of developing CKD. This means that IPA could not only be an important biomarker, but also act as a protective agent against the disease.

## Detection of metabolites using analytical techniques

The detection of renal function is often based on the glomerular filtration rate (GFR). However, it can be challenging to obtain information on CKD aetiology. Moreover, the treatment of CKD in companion animals is often challenging due to possible late diagnosis and progressive renal damage. In order to prevent further development of CKD at the early stages of the disease, metabolites that are accumulated in blood or urine need to be identified. Moreover, metabolites could also provide insight on metabolic pathways and mechanisms that lead to the progression of the disease as well as help to develop therapies needed. This metabolite identification could be carried out by a range of analytical techniques. Table 1 summarises selected metabolites detected in either urine or serum of humans, cats, and dogs with CKD along with identification methods used.

**Table 1:** Examples of detected biomarkers associated with CKD diseases in humans and animals

Metabolite	Metabolites studied (*species*)	Regulation	Expression range (CKD vs healthy)	Method of detection
Albumin	Urine (*dogs*)	Upregulated	26.6 (1.4–228.9) vs 3.1 (± 1.4) mg/dL [13]	SDS-PAGE/MS [13]
Allantoin	Serum (*humans*, *rats*)	Upregulated	27.12 ± 13.78 vs 4.67 ± 2.99 μmol/L [10]	HPLC and UV–Vis (360 nm) [10] Untargeted LC/MS [9] 1H-NMR [24]
Carnosine	Urine (*dogs*)	Upregulated	3.15-fold change CKD compared to healthy group [13]	1D^1^H NMR [13]
Cis-aconitate	Urine (*dogs*)	Upregulated	2.67-fold change CKD compared to healthy group [13]	1D^1^H NMR [13]
Citrate	Serum (*humans*)	Downregulated	754 vs 1060 (nM in 4 mM Creatinine) [5]	1H NMR [21]
Fumarate	Serum (*humans*)	Upregulated	17 vs 12 (nM in 4 mM Creatinine) [5]	Untargeted LC/MS [9]
Symmetric dimethylargine SDMA	Serum (*humans*)	Upregulated	2.05 ± 0.1 vs 0.5 ± 0.04 μmol/L [25]	HPLC [25]
Trigonelline	Urine (*dogs*)	Upregulated	0.15-fold change CKD compared to healthy group [13]	1D^1^H NMR [13]
Uromodulin	Urine (*dogs*)	Downregulated	0 vs 11.9 ± 2.3 mg/dL [13]	SDS-PAGE/MS [13]

Mass spectroscopy (MS) and nuclear magnetic resonance (NMR) are able to detect small molecules in a targeted or untargeted manner. A targeted approach is focused on previously known metabolites, whereas the untargeted approach represents the analysis of all metabolites in a sample [26].

The identification of all metabolites in biofluids remains a challenge. Therefore, a combination of different analytical techniques may be a solution for such blanket identification. Both HPLC (high-pressure liquid chromatography) and GC–MS (gas chromatography-mass spectroscopy) are sensitive techniques, but are also time-consuming as samples require additional preparation [10]. LC–MS (liquid chromatography-mass spectroscopy) possesses high sensitivity and requires minimal sample preparation. However, the drawbacks of LC–MS relate to its high cost and the possibility of sample ion suppression. NMR possesses a number of advantages over these analytical techniques. NMR offers quantification and identification of untargeted metabolites, is non-destructive, and proceeds with rapid data analysis. Moreover, a sample does not require additional preparation for NMR analysis such as protein precipitation, dilution, and solid-phase filtration that is commonly needed for alternative analyses. NMR could be an ideal solution for analysis of urine, plasma, or serum to obtain information on novel or previously detected metabolites associated with CKD. Moreover with the recently demonstrated development of biofluid spectroscopy at low magnetic fields, the near-patient testing potential of NMR is a real possibility [27].

## Conclusion

It is currently a challenge to reliably detect CKD at its early stages in humans and animals. This could lead to mortality and therefore raises clinical concerns. CKD can be detected at the early stages using specific metabolites as biomarkers or signatures. There are a number of a potential biomarkers for CKD have been identified in humans and animals. However, some of these biomarkers need validation across multiple technologies. Moreover, our understanding of biomarkers in animals with CKD is still limited. Analytical techniques such as LC–MS, GC–MS and NMR can provide an insight into the detection of novel metabolites and their concentration associated with the early stages of CKD.
